# Polycystic Ovary Syndrome: Insights into the Therapeutic Approach with Inositols

**DOI:** 10.3389/fphar.2017.00341

**Published:** 2017-06-08

**Authors:** Maria A. Sortino, Salvatore Salomone, Michele O. Carruba, Filippo Drago

**Affiliations:** ^1^Pharmacology Section, Department of Biomedical and Biotechnological Sciences, School of Medicine, Catania UniversityCatania, Italy; ^2^Center for the Study and Research on Obesity, Department of Medical Biotechnology and Translational Medicine, University of MilanMilan, Italy

**Keywords:** polycystic ovary syndrome, insulin resistance, inositol, myo-inositol, D-chiro-inositol

## Abstract

Polycystic ovary syndrome (PCOS) is characterized by hormonal abnormalities that cause menstrual irregularity and reduce ovulation rate and fertility, associated to insulin resistance. Myo-inositol (*cis*-1,2,3,5-*trans*-4,6-cyclohexanehexol, MI) and D-chiro-inositol (*cis*-1,2,4-*trans*-3,5,6-cyclohexanehexol, DCI) represent promising treatments for PCOS, having shown some therapeutic benefits without substantial side effects. Because the use of inositols for treating PCOS is widespread, a deep understanding of this treatment option is needed, both in terms of potential mechanisms and efficacy. This review summarizes the current knowledge on the biological effects of MI and DCI and the results obtained from relevant intervention studies with inositols in PCOS. Based on the published results, both MI and DCI represent potential valid therapeutic approaches for the treatment of insulin resistance and its associated metabolic and reproductive disorders, such as those occurring in women affected by PCOS. Furthermore, the combination MI/DCI seems also effective and might be even superior to either inositol species alone. However, based on available data, a particular MI:DCI ratio to be administered to PCOS patients cannot be established. Further studies are then necessary to understand the real contents of MI or DCI uptaken by the ovary following oral administration in order to identify optimal doses and/or combination ratios.

## Introduction

Polycystic ovary syndrome (PCOS) is a state of hormonal dysregulation and unbalanced ovarian/follicle dynamics affecting 5–10% of women in reproductive age (Asuncion et al., 2000; Azziz et al., 2004). The wide range of PCOS signs and symptoms makes its severity grading challenging. Clinically PCOS can be characterized by some or all of these features: (i) hyperandrogenemia; (ii) oligo/amenorrhoea; (iii) menstrual irregularity; (iv) insulin resistance (IR); (v) presence of 2–9 mm ovarian microcysts; (vi) an ovarian volume greater than 10 ml (The Rotterdam Eshre/Asrm-sponsored PCOS consensus workshop group, 2004). Due to its multifactorial nature, the precise etiology of PCOS has not yet been completely elucidated, but some risk factors (e.g., cardiovascular disease, type 2 diabetes, hypertension and obesity; Orio et al., 2004; Orio and Palomba, 2014) and key triggering conditions (insulin resistance and hyperinsulinemia; Dunaif et al., 1989) have been identified. Given the central role of insulin resistance in the onset of PCOS, insulin-sensitizing agents, such as metformin and pioglitazone, have been proposed as first line approaches (El Hayek et al., 2016). However, the advantage of using these treatments is restricted at lowering IR, while most of women affected by PCOS present also other severe metabolic and reproductive issues (Tang et al., 2012). Such a heterogeneity of clinical manifestations of PCOS suggests that the therapeutic strategy should consider the overall features of the patient and therefore include pharmacological and/or non-pharmacological treatments.

Inositols belong to a sugar alcohol family comprising nine cyclohexane-1,2,3,4,5,6-hexol stereoisomers. These molecules provide the structural basis for inositol phosphates, important secondary messengers in eukaryotic cells, and serve as critical components of the structural lipids, phosphatidylinositol and phosphatidylinositol phosphate (Di Paolo and De Camilli, 2006). Myo-inositol (*cis*-1,2,3,5-*trans*-4,6-cyclohexanehexol, MI) and D-chiro-inositol (*cis*-1,2,4-*trans*-3,5,6-cyclohexanehexol, DCI) represent a promising treatment for PCOS, having shown some therapeutic benefit (El Hayek et al., 2016). Of note, no substantial side effects are reported (El Hayek et al., 2016), although this aspect would probably deserve much attention. In fact, as these compounds are in the market as diet supplements, it is not easy to disclose adverse effects and all the controlled studies that have been carried out, as also discussed below, have usually enrolled too small populations to warrant emergence of significant side effects. Further, not being pharmaceuticals, inositol compounds can be available in many different formulations among countries or geographical areas, making comparison between clinical trials conducted in different locations difficult. However, Carlomagno and Unfer (2011) reported the high safety of MI examining different studies in which the compound was used at doses up to 18 g/day, which was the maximum tolerated dose (Lam et al., 2006). The most frequently observed adverse effects were gastrointestinal with diarrhea, loose stool, flatulence, and nausea. Mild insomnia was occasionally reported (Carlomagno and Unfer, 2011). Because the use of inositols for treating PCOS is widespread, a deep understanding of this treatment option is needed, both in terms of potential mechanisms and efficacy. This issue is of particular interest, considering the economic and social burden of PCOS (Azziz et al., 2005), which not only represents a leading cause of female infertility worldwide (Conway et al., 2014; Joham et al., 2015), but has also been associated with a higher risk of ovarian cancer (Lauretta et al., 2016).

Thus, the aim of this review is to give an overview of PCOS and to summarize the current knowledge on the biological effects of MI and DCI; in the second part, the results obtained from relevant intervention studies will be critically discussed.

## PCOS: Overview of a Complex Disease

### Pathophysiology

The pathophysiology of PCOS is considered multifactorial, involving genetic, environmental and metabolic abnormalities (El Hayek et al., 2016). More recently, however, increasing evidence supports the role of IR. Among the genetic causes, mutations in genes involved in the synthesis, transport and regulation of androgens have been pointed out (Urbanek, 2007). Other genes that may be involved in this syndrome are those regulating gonadotropin signaling, phosphatidylinositol-3-kinase (PI3-kinase) activation, glucose transporter 4 (GLUT4) expression, and DNA repair pathways (Chen et al., 2011; Du et al., 2014; De Leo et al., 2016). However, due to the small size of populations studied and to the fact that, often, several genes are simultaneously involved in the pathogenesis of PCOS, the genetic basis of PCOS remains largely to be understood (Franks et al., 1997; Urbanek, 2007). Regarding exogenous factors involved in PCOS onset, it has been found that the prevalence of PCOS is similar in most countries, but specific cultural and environmental factors, such as diet, physical activity, and life-style, may influence the phenotypic manifestations of the syndrome (De Leo et al., 2016). More recently, the pro-inflammatory status caused by oxidative stress (OS) has been associated to IR, hyperandrogenism, and PCOS (Victor et al., 2009; Murri et al., 2013). Hyperglycemia in itself might sustain an increase in the production of reactive oxygen species (ROS) by peripheral blood leukocytes, which in turn would impact on several pathways in ovary, including oocyte maturation, ovarian steroidogenesis, corpus luteum functions, and embryo development (González S. et al., 2012; González F. et al., 2012). Consistently, increased concentration of OS markers in the follicular fluid from PCOS patients has been reported (Bausenwein et al., 2010; Chattopadhayay et al., 2010). Mitochondrial dysfunction is in fact implicated in PCOS development, with reduced mitochondrial mass, and impaired expression and activity of several mitochondrial proteins (Victor et al., 2015). Notably, mitochondrial deficits have been found in metabolically active tissues, such as adipose and muscle tissues, of obese and diabetic subjects (Valerio et al., 2006; Nisoli et al., 2007). Moreover, adipose pro-inflammatory cytokines, including TNF-α, overexpressed in obese patients, was found to down-regulate endothelial nitric oxide synthase (eNOS) expression and, consequently, nitric oxide (NO) production (Valerio et al., 2006; Nisoli et al., 2007). The reduced NO levels in turn impair mitochondrial biogenesis and function of adipocytes and muscle cells (Nisoli et al., 2007). Accordingly, two relevant morbidities most often associated with PCOS are obesity and diabetes (Kulshreshtha et al., 2013; El Hayek et al., 2016), with a prevalence of ∼50 and ∼27% (Peppard et al., 2001), respectively, in the PCOS population. In particular, childhood obesity is a well-documented risk factor for PCOS and obese girls have higher risk of developing IR and PCOS (Pasquali et al., 2011); on the other hand, women with PCOS are at a higher risk of developing obesity (El Hayek et al., 2016). Increased androgen levels have been found in women with upper body obesity (Kirschner et al., 1990), while women with PCOS have a masculinized body fat distribution, with increased visceral/subcutaneous body fat (Borruel et al., 2013), which correlates with the degree of IR (Karabulut et al., 2012). Obese women affected by PCOS present also other metabolic alterations, such as elevated levels of low-density lipoproteins, triglycerides and cholesterol, along with decreased levels of high-density lipoproteins (El Hayek et al., 2016), which make them at high risk of developing cardio-vascular diseases. PCOS also substantially increases the risk of developing type 2 diabetes mellitus, mainly due to the associated obesity and IR (Peppard et al., 2001). Furthermore, in families of PCOS patients the prevalence of type 2 diabetes mellitus is increased (Dunaif et al., 1989; El Hayek et al., 2016).

### The Key Role of Insulin Resistance

**FIGURE 1:**
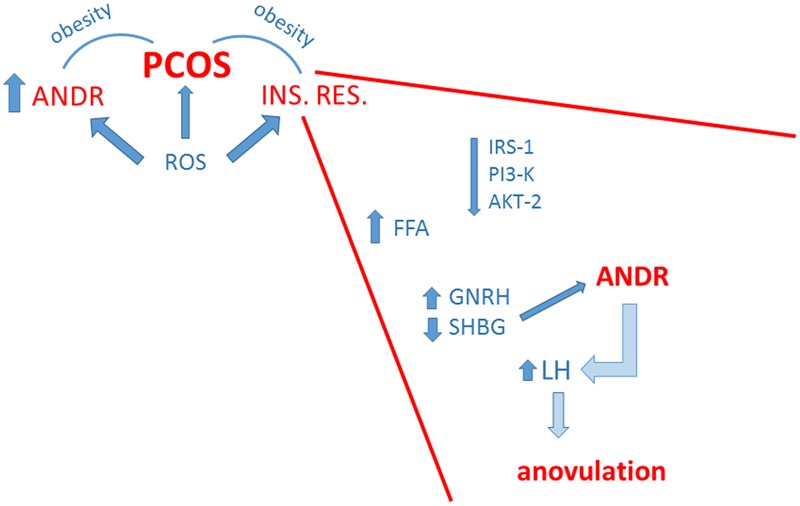
Schematic summary of the strict correlation among polycystic ovary syndrome (PCOS), insulin resistance (INS. RES.) and hyperandrogenemia (ANDR). The enhanced production of radical oxygen species (ROS) contributes to all these conditions. Obesity linked to hyperandrogenemia and/or insulin resistance concurs to PCOS. In the inset, major factors at the basis of insulin resistance are depicted. These include general weakening of insulin signaling, but also metabolic and hormonal dysregulations that favor hyperandrogenemia with consequent impairment of ovulatory function.

Insulin resistance is a condition in which cells, tissues or a whole organism do not regularly respond to insulin, requiring higher amounts of the hormone to obtain the expected biological effect. This circumstance causes, in turn, a phenomenon called “compensatory hyperinsulinemia” with an increase of insulin secretion by the pancreatic β-cells while the blood glucose levels remain in the normal range, causing a vicious circle and predisposing the patient, when the response of pancreatic cells decreases, to the onset of glucose intolerance or type 2 diabetes. As previously stated, IR plays a central role in approximately 70–80% of obese women and in 15-30% of lean women diagnosed with PCOS (Fauser et al., 2012), and represents the pathogenic link between metabolic and reproductive disorders in PCOS. The decrease in insulin sensitivity has been attributed to post-receptor alterations in intracellular signaling pathways of insulin occurring in PCOS (Højlund, 2014); different studies have indeed detected defects in insulin signaling through insulin receptor substrates (IRS-1; Langlais et al., 2011), Akt2 (Tan et al., 2007), PI3K (Cusi et al., 2000), and AS160/TBC1D4 (Larance et al., 2005), which can account for reduced insulin action on glucose transport. Another feature linked to insulin resistance is a rise in free fatty acid (FFA) plasma levels. This condition could be due to an increased synthesis and mobilization from liver and adipose tissue, respectively. The excess of FFA leads *per se* to IR by decreasing the activity of key enzymes such as pyruvate dehydrogenase or by decreasing glucose transport activity (Dresner et al., 1999). IR is associated to hyperandrogenism, menstrual irregularities, and other metabolic manifestations of PCOS (Baillargeon et al., 2003). Hyperandrogenism triggers an excessive production of acyclic estrone which, in turn, determines an overproduction of gonadotropins, especially luteinizing hormone (LH; Højlund, 2014). The elevated levels of circulating insulin in women with PCOS, together with high levels of LH, could arrest follicular growth, contributing to the onset of an anovulatory phase (De Leo et al., 2016; El Hayek et al., 2016). Hypersecretion of LH in these women may also promote early luteinization of granulose cells and early arrest of antral follicle development (Piouka et al., 2009; Liu et al., 2012; Cadagan et al., 2016). Moreover, LH may activate premature meiotic processes that damage oocyte quality, thereby contributing to embryonic aneuploidies (Qiao and Feng, 2011). Hyperinsulinemia leads to alteration of the secretion of gonadotropin-releasing hormone (GnRH) and to inhibition of the hepatic synthesis of sex hormone-binding globulin (SHBG), leading to increased concentration of circulating free androgens (Toprak et al., 2001). Other factors can concur to cause hyperandrogenism, such as an excess of androgen synthesis in the adrenal gland and the presence of enzymatic defects of ovarian and adrenal steroidogenesis (Genazzani et al., 1993). These mechanisms are summarized in **Figure 1**.

Women with PCOS have higher concentrations of anti-mullerian hormone (AMH) in serum and follicular fluid, which strictly correlate with the arrest of follicular growth and with testosterone and/or LH concentrations (Homburg et al., 2013; De Leo et al., 2016).

## Inositols: Physiological Roles

### Biological Functions

**FIGURE 2:**
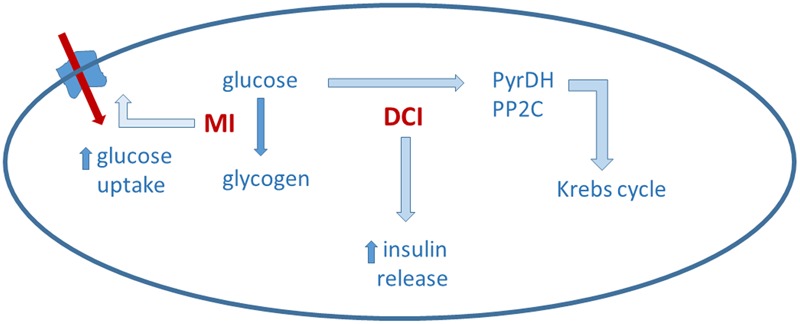
Myo-inositol (MI) and D-chiro-inositol (DCI) may act in a complementary way on glucose metabolism. Details in the text.

Inositols are present in cells both as free form and as components of membrane phosphoinositides and take part to a great variety of functions, including cell growth and survival, development and function of peripheral nerves, osteogenesis and reproduction (Di Paolo and De Camilli, 2006). In their conjugated form, inositols are components of cellular membranes and have a crucial function in membrane integrity and in intracellular signaling (Di Paolo and De Camilli, 2006). Phosphatidylinositol is the precursor of phosphatidylinositol phosphate and phosphatidylinositol diphosphate (PIP2), which upon hydrolysis by phospholipase C (PLC) gives inositol 1,4,5 trisphosphate; this latter acts as second messenger of membrane receptors coupled to PLC, being involved in the signaling mechanism of many autacoids, hormones, and neurotransmitters (Di Paolo and De Camilli, 2006). Both MI- and DCI-phosphoinositides are able to influence the intracellular metabolic processes activating key enzymes involved in oxidative and non-oxidative glucose metabolism (Larner et al., 1988; Lauretta et al., 2016). MI is involved in the metabolism, transport and breakdown of glucose and its conversion to glycogen (Croze and Soulage, 2013), while DCI is involved in the insulin-signaling pathway and in the stimulation of serial enzymes that are in turn involved in the regulation of glucose metabolism, (e.g., pyruvate dehydrogenase phosphatase (PDHP), protein phosphatase 2C (PP2C), inositol-phosphate glycan; Larner, 2002). Furthermore, it has been suggested that MI and DCI work in synergy in the glucose metabolism; in particular, MI induces the translocation of glucose transporter to the cell membrane thereby enhancing glucose cellular uptake (Yap et al., 2007; Dang et al., 2010), while DCI stimulates pyruvate dehydrogenase and supports ATP production via the Krebs’ cycle (Larner, 2002). Moreover, recent data indicate that DCI glycans specifically stimulate insulin secretion in pancreatic β-cells (Lazarenko et al., 2014). These hypothetical mechanisms are summarized in **Figure 2**.

Insulin signaling typically involves insulin receptor tyrosine kinase that autophosphorylates and then phosphorylates IRS. One of the main target of IRS is PI3K, which is involved in the phosphorylation of Akt, a protein kinase that finally leads to an increase in the translocation of glucose transporter into the plasma membrane (Taniguchi et al., 2006). In addition to this, it has also been proposed that insulin binding to its receptors induces the production of an inositol glycan second messenger, termed INS-2 that, once released, activates PP2Cα and mitochondrial PDHP. In the cytosol, activated PP2Cα would stimulate glycogen synthase, while in the mitochondria, PDHP would induce pyruvate dehydrogenase, which promotes glucose oxidative use (Larner et al., 2010; Croze and Soulage, 2013). This hypothesis, proposed by Larner (2002), is mostly based on the idea that insulin receptor may signal also through G_q/11_, coupled to a PLC, and relies on data showing co-localization of G_q/11_ with the insulin receptor in membrane vesicles (Sleight et al., 2002). Activation of G_q/11_ by insulin receptor, however, has not been yet firmly demonstrated.

### Dietary Uptake, Tissue Distribution and Metabolism

Following ingestion with the diet, adsorbed inositols reach plasma/interstitial fluids and are subsequently taken up by tissues and cells by a membrane dependent sodium-inositol cotransporter (SMIT1/2; Coady et al., 2002; Bourgeois et al., 2005). Some tissues (liver, nervous system) seem to actively concentrate MI, leading to significant differences in inositol content among different tissues (Greene and Lattimer, 1982; Prpić et al., 1982; Kollros et al., 1990; Uldry et al., 2001). In cultured human cells, MI transport ensured by SMIT1/2 increases following down regulation of protein kinase C activity and decreases following activation of a protein kinase A, suggesting that the inositol uptake system is post-translationally regulated through phosphorylation (Preston et al., 1995). In diabetic patients, MI excreted with urines is more abundant than in healthy volunteers, due to competition of glucose with MI tubular transport (Daughaday and Larner, 1954).

Biosynthesis of MI occurs also endogenously, primarily in the kidney, with a rate approaching 4 g/day. Extra renal tissues (e.g., brain, testis, and liver) can also contribute to the production of inositol under hormonal control (Hasegawa and Eisenberg, 1981). Kidney is the only organ of relevance in inositol catabolism, given that nephrectomy in *in vivo* models impairs MI degradation, while renal failure has been associated with significant abnormalities in MI metabolism and increased plasma levels of inositol (Pitkänen, 1976). Inositol is synthesized from glucose-6-phosphate (G6P) through two biochemical reactions: G6P is first isomerized by the NADH-dependent, cytosolic D-3-MI-phosphate synthase (INO1 or MIPS1, encoded by ISYNA1 gene) to inositol-3-phosphate, which is then dephosphorylated by inositol monophosphatase-1 (IMPA-1) to yield free MI (Loewus et al., 1980). MI is converted into DCI by an NAD/NADH epimerase (Pak et al., 1992). The conversion rate of MI to DCI ranges from 7% to about 9%, as measured by the analysis of radiolabeled [^3^H]-MI, whereas the production of other isomers is minimal, not exceeding 0.06% of total radiolabeled MI (Pak et al., 1992). In muscles of patients suffering from IR conversion of MI to DCI is reduced due to a decreased epimerase activity (Sun et al., 2002). Women affected by PCOS also show reduced serum levels and increased urinary loss of DCI (Baillargeon et al., 2006, 2008). It has been also reported that the urinary clearance of DCI is inversely correlated to insulin sensitivity in PCOS women, representing a strong and independent predictor of IR (Baillargeon et al., 2006, 2008). These data support the hypothesis that PCOS patients, due to IR, experience a severe dysregulation of inositol metabolism, characterized by an imbalance, with an excess of MI and a deficiency of DCI, together with a reduction of MI/DCI epimerase activity. Such an imbalance has been observed in obese PCOS patients, while data in lean PCOS patients are not available (Baillargeon et al., 2010). However, because the beneficial effect of inositols in reducing IR in intervention studies are not correlated with BMI values (**Table 1**), it is likely that such a dysregulation also occurs in lean women.

**Table 1:** Intervention studies with myo-inositol (MI), D-chiro-inositol (DCI) and a combination of DCI and MI in women affected by PCOS.

						Outcomes		
	Reference	Study design	Treatment	N. of subjects	BMI	Hormonal	Insulin Resistance	Reproductive
**MI**					
	Artini et al., 2013	RCT	MI 2 g/day + FA 200 mg/day No other drug treatment	50	26.5 ± 6.1	Reduction of plasma LH, PRL, T, insulin, and LH/FSH	Reduction	NA
	Ciotta et al., 2011	RCT	MI 2 g/twice a day + FA 200 mcg/twice a day COH	34	NA	Reduction in total rFSH units and in E2	NA	Increase number of mature oocytes; higher mean number of transferred embryos
	Costantino et al., 2009	RCT	MI 4 g/day + FA 400 mcg/day No other drug treatment	42	22.8 ± 0.3	Decrease of serum free T	Reduction; improvement of glucose tolerance and reduction of glucose stimulated insulin release	NA
	Genazzani et al., 2008	RCT	MI 2g/day + 2 mcg FA No other drug treatment	20	29 ± 1.6	Reduction in LH, PRL, T, insulin, and LH/FSH	Reduction	Restoration of menstrual cyclicity
	Genazzani et al., 2012	Observational	MI 2 g/day No other drug treatment	42	31.1 ± 1.4	Reduction of LH, LH/FSH, and insulin	Reduction	NA
	Gerli et al., 2007	RCT	MI 4 g/day + FA 400 mcg/day No other drug treatment	92	34.0 (CI: 31.5–36.5)	Increase of E2	No change in fasting glucose concentrations, fasting insulin, or insulin responses to glucose challenge	Higher ovulation frequency, shorter time to first ovulation and more rapid follicular maturation
	Minozzi et al., 2008	Open-label	MI 2 g/twice a day No other drug treatment	46	27.5	Reduction of total androgens, FSH and LH; increase of oestradiol	Reduction	NA
	Minozzi et al., 2011	Prospective open-label	OCP + MI 4 g/day No other drug treatment	155	26.7 ± 2.7	Reduction of androgens	Reduction	NA
	Pacchiarotti et al., 2016	RCT	Group A: MI 4 g/day + FA 400 mcg/day + melatonin 3 mg/day Group B: MI 4 g/day + FA 400 mcg/day COH	526	22.8 ± 1.3	Less total gonadotropin dose administered in group A versus B	NA	Increased number of mature oocytes and increased percentage of grade I embryos in group A versus group B
	Papaleo et al., 2007	RCT	MI 2 g/twice a day + FA 400 mcg/day No other drug treatment	25	28.5 ± 2.4	NA	NA	Restoration of menstrual cyclicity, increase rates of pregnancies
	Raffone et al., 2010	RCT	MI 4 g/day + FA 400 mcg/day or metformin 1500 mg/day	120	25 ± 2.1	NA	Reduction	Restored spontaneous ovulation activity and menstrual cycle
	Rago et al., 2015	Pilot study	MI + α-LA IVF	65	NA	NA	Reduction	Number of transferred embryos Clinical pregnancy
	Sacchinelli et al., 2014	Prospective	MI 4 g/day + NAC + FA 400 mcg/day No other drug treatment	91	29.2 ± 2.21	NA	Reduction of HOMA	Increase in ovulation

**DCI**					
	Cianci et al., 2015	Prospective	DCI 1 g/day + LA 600 mg/day No other drug treatment	46	28.7 ± 2	Reduction of insulin	Reduction	Restoration of menstrual cyclicity
	Genazzani et al., 2014	Observational	DCI 500 mg/day No other drug treatment	22	31.5 ± 0.8	Improvement in LH, LH/FSH, androstenedione, GnRH	Reduction	NA
	Iuorno et al., 2002	Observational	DCI 600 mg/day No other drug treatment	20	22.4 ± 0.3	Decrease of serum T	Decrease of the insulin AUC	Restored ovulation activity
	La Marca et al., 2015	Retrospective	DCI 1-1.5 g/day No other drug treatment	47	23 ± 4.1	Reduction of AMH	Reduction	Increase in regular menstrual cycles
	Laganà et al., 2015	Observational	DCI 1 gr/day + FA 400 mcg/day No other drug treatment	48	24.87 ± 5.21	Reduction of LH, LH/FSH ratio, total and free T, ?-4-androstenedione and increase of SHBG	Reduction of HOMA and increase of glycemia/IRI ratio	Restoration of menstrual cyclicity
	Nestler et al., 1999	RCT	DCI 1.2 g/day No other drug treatment	44	31.3 ± 2.4	Decrease of serum free T	Decrease of insulin AUC	Restored ovulation activity
	Piomboni et al., 2014	RCT	Group A: DCI 1g/day + COH Group B: metformin 1.7 g/day + COH	68	25.2 ± 4.1	NA	NA	Improved oocyte quality

**MI+DCI**					
	Colazingari et al., 2013	RCT	Group A: MI 550 mg/twice day + DCI 13.8 mg/twice a day Group B: DCI 500 mg/twice a day COH	100	<28	Reduction in total rFSH units and in peak E2 levels	NA	Group B: increase number and quality of oocytes. Group A: higher fertilization rate and embryo quality; greater number of transferred embryos.
	Minozzi et al., 2013	Longitudinal	MI 550 mg + DCI 13.8 mg twice a day No other drug treatment	20	33.71 ± 6.1	Improvement in LDL, HDL, and triglycerides levels	Reduction	NA
	Nordio and Proietti, 2012	RCT	Group A: MI 2 g/twice a day Group B: MI 550 mg/twice a day + DCI 13,8 mg/twice a day	50	27.5 ± 2.9	Reduction of total T and increment of SHBG higher in group B compared to A	Reduction in glucose and insulin levels in group B	Restored ovulation activity

*AMH, anti-müllerian hormone; AUC, area under the curve; BMI, body mass index (in the treated group, before starting the treatment); COH, controlled ovarian hyperstimulation (GnRH agonist, rFSH, hCG); E2, estradiol; FA, folic acid; FSH, follicle-stimulating hormone; GnRH, gonadotropin-releasing hormone; IRI, immune reactive insulin; IVF, *in vitro* fertilization; HDL, high-density lipoprotein; HOMA, homeostatic model assessment; LA, lipoic acid; LDL, low-density lipoprotein; LH, luteinizing hormone; NA, not available; NAC, *N*-acetylcysteine; OCP, oral contraceptive pill (estradiol 30 μg/gestodene 75 μg); PRL, prolactin; RCT, randomized control trial; rFSH, recombinant follicle-stimulating hormone; SHBG, sex hormone binding globulin; T, testosterone.*

However, it has been proposed, that, in women affected by PCOS, ovaries remain insulin sensitive, in contrast to most other tissues and organs, (Carlomagno et al., 2011, 2015; Di Nicola et al., 2014; Unfer and Porcaro, 2014). Actually, this speculation is based on data from only one study, reporting higher MI/DCI epimerase activity in theca cells from PCOS compared to control women (Heimark et al., 2014). In this study, mean values of MI/DCI epimerase activity in PCOS were about three times higher than in controls, while the MI/DCI ratio was 3–4 times higher in controls than in PCOS. Other authors have examined the content of MI and DCI in follicular fluid, by using a vaginal probe; they report a MI:DCI ratio equal to 0.2:1 in PCOS and to 100:1 in healthy women (Unfer et al., 2014). There appears to be some inconsistency between the two studies, considering a threefold difference in the MI:DCI ratio as measured in cultured theca cells, versus a 500-fold difference in the MI:DCI ratio, as measured in the follicular fluid; on the other hand, a robust literature reports *in vivo* data providing evidence of peripheral IR in PCOS associated to decreased epimerase activity and increased MI to DCI ratios (Kennington et al., 1990; Asplin et al., 1993; Suzuki et al., 1994; Larner and Craig, 1996; Pak et al., 1998; Sun et al., 2002). Thus, additional studies are warranted to ascertain whether or not a greater epimerase activity, leading to a decreased MI/DCI ratio, occurs in the ovary of women affected by PCOS.

### Inositol’s Effects in PCOS

The rationale behind the use of inositols in PCOS derives from studies showing that PCOS patients, due to IR, have an imbalance, with an excess of MI and a deficiency of DCI, together with a reduction of MI/DCI epimerase activity in peripheral tissues. It is, however, not known at the molecular and cellular level whether inositol supplementation directly affects insulin signaling and/or restores insulin sensitivity. Such an effect would be beneficial as both IR and secondary hyperinsulinemia trigger hyperandrogenic anovulation and/or irregular cycles. Up to now, evidence supporting beneficial clinical effects of inositol supplementation in PCOS is provided, but mechanisms underlying these effects have not been identified. **Table 1** summarizes results of intervention studies with MI, DCI, and a combination of DCI and MI in women affected by PCOS.

While a number of studies have analyzed the effectiveness of MI and DCI, alone or in combination, in PCOS (see below), only two studies have compared the effects of MI to DCI and found that they seem to exert comparable effects. The first study compared two groups, daily-treated for 6 months with either 4 g MI plus 400 μg folic acid or with 1 g DCI plus 400 μg folic acid. The results indicated that both MI and DCI were effective in improving ovarian function and metabolism in patients with PCOS, but MI showed a more marked effect on the metabolic profile, whereas DCI was more effective in reducing hyperandrogenism (Pizzo et al., 2014). The second study compared the clinical and metabolic response after 6 months of therapy with MI or DCI or placebo in 137 PCOS women. Results indicated that both MI and DCI were equally able to improve the regularity of the menstrual cycle, the Acne Score, the endocrine and metabolic parameters and the insulin resistance (Formuso et al., 2015).

### Clinical Evidence of MI Effectiveness

Several studies on women affected by PCOS have shown that MI supplementation can improve menstrual regularity and IR, while reducing hyperandrogenism (Papaleo et al., 2007; Genazzani et al., 2008, 2012; Minozzi et al., 2008; Ciotta et al., 2011; Unfer et al., 2012; Croze and Soulage, 2013). Regarding the improvement of both hormonal and insulin resistance, the efficacy of 2 g MI for 8 weeks of treatment has been investigated in 42 PCOS obese women and significantly improved these parameters in all of them. Interestingly, a sub-set analysis of PCOS women with fasting insulin levels above 12 μU/mL revealed that they experienced a greater reduction of both fasting insulin plasma levels and area under the curve (AUC) of insulin under OGTT compared to patients with fasting insulin levels below 12 μU/mL (Genazzani et al., 2012). As mentioned above, MI, has also been shown to decrease hyperandrogenism in women with PCOS (Minozzi et al., 2008; Croze and Soulage, 2013). In particular, Minozzi and colleagues, enrolled 46 hirsute women which were given 2 g MI therapy for 6 months. Even if no changes in body mass index (BMI) were observed, hirsutism decreased after therapy, as well as total androgens, FSH and LH, while estradiol increased; IR, analyzed by the homeostatic model assessment (HOMA), was also significantly reduced. A meta-analysis has reviewed six Randomized Controlled Trials (RCTs) that assessed the effectiveness of MI supplementation in PCOS, reporting that the higher dose of 4 g MI/day for 12 and 16 weeks seems to achieve better results than lower doses (**Table 1**); however, MI doses used in most published studies range from 2 to 4 g/day (Unfer et al., 2012). In general, no side effects have been reported and overall results provide level IA evidence of MI effectiveness, mainly assessed as improved insulin sensitivity (Nestler et al., 2012).

Several studies have analyzed the combined administration of MI and folic acid in PCOS patients. Papaleo et al. (2007) have reported that 2 g MI plus 200 μg of folic acid twice a day for 6 months restored spontaneous ovarian activity and fertility in patients with PCOS. In these patients, MI induced the maintenance of normal ovulatory activity in 72% of cases, with a pregnancy rate of 40% during the 6-month observation period (Papaleo et al., 2007). In another study, a double blind trial, patients received 2 g of MI plus 200 μg folic acid or 200 μg folic acid only, twice a day, for 3 months. At the end of treatment, in the MI group there was a higher number of follicles with diameter > 15 mm, visible at ultrasound during stimulation and more oocytes were collected (Ciotta et al., 2011).

A RCT was also performed in 20 overweight PCOS women that received 2 g of MI plus 200 μg folic acid or 200 μg folic acid only. Patients taking MI experienced an improvement of reproductive axis and IR state after 12 weeks of supplementation, while no change occurred in patients treated only with folic acid (Genazzani et al., 2008). In details, all patients underwent hormonal evaluations and an OGTT before and after therapy; ultrasound examinations and Ferriman–Gallwey score were also performed. After 12 weeks MI administration plasma LH, PRL, T, insulin levels and LH/FSH ratio were significantly reduced and insulin sensitivity, expressed as glucose-to-insulin ratio and HOMA index, resulted significantly improved. Menstrual cycle was restored in all subjects with amenorrhea or oligomenorrhea (Genazzani et al., 2008). Positive effects of MI on ovarian function have been highlighted also in another RCT examining 50 overweight PCOS patients in which group A was given 2 g MI plus 200 μg folic acid daily for 12 weeks and group B was administered only 200 μg folic acid daily. The Authors found significant improvement in hormonal parameters and restoration of menstrual cycle in all patients with amenorrhea and oligomenorrhea belonging to group A, while no changes were noted in group B patients (Artini et al., 2013). Moreover, reduced plasma LH, prolactin, LH/FSH ratio, and IR measured by HOMA index were observed in the MI group. In another recent study, 50 anovulatory PCOS patients with IR were given 2 g MI and 200 μg folic acid for three cycles, twice a day. Ovulation and pregnancy were achieved in 61.7 and 37.9% of women, respectively. In women remaining anovulatory, MI was subsequently used in combination with clomiphene citrate for three cycles resulting in ovulation and pregnancy rates of 72.2 and 42.6%, respectively. These patients had also a reduction in BMI and HOMA index (Kamenov et al., 2015). A significant weight reduction along with a decrease in leptin levels has been also reported in a double blinded, placebo-controlled study in which 92 women were randomized to receive 400 μg folic acid or 4 g MI plus folic acid for 14 weeks (Gerli et al., 2007). Interestingly, addition of α-lipoic acid to MI in PCOS women produced a stronger reduction of BMI, insulin levels and ovarian volume when compared to MI alone (Rago et al., 2015). However, reproductive outcomes, including number of transfered embryos and clinical pregnancy did not differ between treatment groups (Rago et al., 2015).

### Clinical Evidence of DCI Effectiveness

As previously mentioned, women with PCOS (Kennington et al., 1990; Baillargeon et al., 2006, 2008) and type II diabetes (Hong et al., 2012) have reduced serum level and increased urinary loss of DCI. This could be due to multiple causes, including a defective conversion of MI to DCI and/or to impairment of tubular transport by high glucose. Administration of DCI could reestablish an adequate tissue content of DCI derivatives, increase insulin sensitivity and improve ovulatory frequency and serum androgens and/or levels of lipid biomarkers in women affected by PCOS (Nestler et al., 1999; Iuorno et al., 2002; Kawa et al., 2003; Cheang et al., 2008). Those effects are mainly ascribed to a DCI systemic activity, able to counteract the main consequences of the metabolic syndrome that are associated with PCOS (Gerli et al., 2003). A very recent study shows that DCI is able to reduce the expression of CYP19A1 genes, P450scc and insulin-like growth factor 1 receptor (IGF-1R) in a dose-response manner, contrasting the up-regulation of enzymes involved in steroidogenesis, thus, confirming its role as a modulator of insulin levels in the ovary (Sacchi et al., 2016).

A randomized, double-blind controlled trial was conducted on 22 obese women affected by PCOS (Nestler et al., 1999). Patients received 1.2 g DCI or placebo for 6–8 weeks. The results showed a decrease of the mean insulin AUC following oral administration of glucose, as well as of serum free testosterone, plasma triglycerides, diastolic and systolic blood pressure. Furthermore, 19/22 women who received DCI ovulated, as compared with 6/22 in the placebo group (Nestler et al., 1999). Favorable results have also been recently reported by Genazzani et al. (2014) who conducted a study on a group of overweight/obese PCOS patients (BMI > 26) receiving 500 mg/day DCI, for 12 weeks. After the treatment, LH, FSH, androstenedione and insulin levels improved as well as GnRH-induced LH response. BMI decreased, though no lifestyle modification was requested. Interestingly, it was shown that DCI was particularly effective in PCOS women with a family history of diabetes (Genazzani et al., 2014). Similar effective results were obtained in 20 lean women (BMI, 20.0–24.4 kg/m^2^) with PCOS treated with 600 mg DCI, once daily for 6–8 weeks. In DCI-treated patients the mean insulin AUC after oral administration of glucose decreased, as well as serum free testosterone, systolic and diastolic blood pressure, and plasma triglyceride concentrations. Data from ovulation were not statistically significant (Iuorno et al., 2002). Further evidence that DCI administration to PCOS patients is able to improve insulin sensitivity and to reduce serum free testosterone levels, leading to normal cycle and ovulation are available from a retrospective study performed in PCOS patients with irregular cycles. In this case 1–1.5 g DCI administered daily for a maximum of 15 months improved insulin levels along with an increase in the percentage of women reporting regular menstrual cycles, directly proportional to the duration of the treatment (24 and 51.6% at a mean of 6 and 15 months of treatment; La Marca et al., 2015). Moreover, DCI administration modulated the secretion of AMH (La Marca et al., 2015). In another study, 1 g DCI plus 400 μg folic acid daily for 6 months significantly improved IR as measured by HOMA index and glycaemia/insulin resistance index (IRI) ratio. In the same study an improvement of systolic blood pressure, Ferriman–Gallwey score, LH, LH/FSH ratio, total testosterone, free testosterone, Δ-4-androstenedione, prolactin, and sex hormone binding globulin were observed (Laganà et al., 2015). Despite this large body of evidence showing positive effects of DCI in the treatment of PCOS, Legro (2016) reported that large, multicenter phase 2 clinical trials were suspended because of lack of efficacy.

Considering that oxidative stress may have a significant impact on IR and metabolic profile, recent studies are analyzing the effect of DCI administration, alone or in combination with antioxidants, on oxidative stress. To evaluate the effects of the combination of DCI and alpha lipoic acid on PCOS metabolic disorders, 46 women (26 study group, 20 controls) at reproductive age, with PCOS according to Rotterdam criteria (The Rotterdam Eshre/Asrm-sponsored PCOS consensus workshop group, 2004), were analyzed in a prospective study at baseline and after 180 days. Clinical and metabolic aspects of women on DCI and lipoic acid treatment underwent improvement with respect to the control group, while no statistically difference was observed in lipid profile (Cianci et al., 2015). Another study evaluated the oxidative stress status following gonadotropin administration, alone or in combination with DCI or metformin, by measuring amino acidic free-SH groups and by checking the oocyte quality according to international morphological criteria in women with PCOS (Piomboni et al., 2014). Both DCI and metformin decreased OS level, while a higher number of good quality oocytes was observed in the DCI group in comparison to the control group (Piomboni et al., 2014).

### Clinical Evidence of Effectiveness of MI/DCI Combinations

Different authors examined the possibility of administering the MI/DCI combination (Nordio and Proietti, 2012; Colazingari et al., 2013; Unfer and Porcaro, 2014). Based on the supposed alteration of MI/DCI ratio occurring in ovary in PCOS, the combination MI/DCI 40:1 was tested. Two small studies from the same group (Nordio and Proietti, 2012; Minozzi et al., 2013) investigated a combination of MI plus DCI containing 550 mg MI + 13.8 mg DCI (equivalent to 3300 mg MI + 84 mg DCI in powder format). The first study compared the effects of MI plus DCI treatment with that of a dose of 4 g MI/day in powder form. They found that, after 6 months of treatment, both MI and MI plus DCI groups showed improvements in various metabolic markers of PCOS, but the combined MI/DCI resulted twice as effective in reducing HOMA-IR, compared to MI alone (Nordio and Proietti, 2012). The second study enrolled 20 obese women with PCOS and analyzed the lipid profile before and after treatment (6 months) with 550 mg MI + 13.8 mg DCI. Results showed that the combination therapy induced an improvement, compared with placebo, of LDL, HDL, and triglycerides (Minozzi et al., 2013). A single study compared the combination MI/DCI (40:1) to treatment with DCI only in PCOS women undergoing *in vitro* fertilization (IVF). The patients were treated twice a day with either 1.1 g MI plus 27.6 mg DCI or with 500 mg DCI, for 12 weeks before rFSH administration and throughout pregnancy. The results suggest that combination therapy improved oocyte and embryo quality and the chance of becoming pregnant (Colazingari et al., 2013). However, these beneficial effects occurred in the younger (<35 years) but not in the older (>35 years) population.

These preliminary data suggest that the combination MI/DCI might be more effective than the supplementation with a single inositol species, but because of the small number of treated patients they need to be confirmed in larger studies. The MI/DCI ratio chosen for the treatment with the combination seems arbitrary because, while an imbalance in the MI/DCI ratio might be present in the PCOS ovary, possibly due to enhanced epimerase activity (Heimark et al., 2014; Unfer et al., 2014), the inositol plasma levels are quite different; for example a study by Baillargeon et al. (2006) reports a 111 MI/DCI ratio in healthy individuals and a 206 MI/DCI ratio in PCOS patients, i.e., a twofold relative increase in circulating MI, compared to a 500-fold relative decrease supposedly occurring in the ovary (see also above); such plasma inositol levels are related to an increased urinary clearance of DCI in PCOS, without changes in MI clearance. Hence, without further scientific evidence, the MI:DCI at 40:1 ratio has no reason to be considered preferable to other available formulations.

Because any oral treatment reaches the plasma compartment before diffusing to target tissues, including ovary, the MI/DCI ratio to be used in a combination for treating PCOS should chiefly take into account the plasma AUC it produces for the two inositols. Starting from the alteration reported in inositol plasma concentration (a DCI reduction with MI barely affected), the supplement treatment should aim at restoring the normal MI and DCI concentrations; as a consequence, ovary inositol uptake and concentrations might conceivably be restored, but this should be directly assessed in *ad hoc* studies. In the absence of MI and DCI plasma AUCs following oral administration of MI/DCI combinations, the efficacy of a given combination can be predicted only on empirical basis. Finally, based on the reported epimerase activity in the PCOS ovary (Heimark et al., 2014) a treatment strategy using an overwhelming amount of MI over DCI (such as with the ratio 40:1) could exert paradoxical effects because the efficient conversion of MI to DCI could further increase the MI/DCI imbalance.

## General Conclusion

Polycystic ovary syndrome is a common endocrine and metabolic disorder characterized by oligo-anovulation, hyperandrogenism, and insulin resistance, this latter playing a key role in the pathogenesis of this syndrome, both in lean and obese women. Various therapeutic approaches have been attempted in PCOS, including supplementation with inositols, MI and DCI, which may have distinct and synergic physiologic roles in the metabolism of glucose and in the regulation of insulin action, counteracting the endocrine disorder of this syndrome. Because inositols are natural, endogenous compounds, they can be considered as a safe treatment for PCOS patients. However, the precise cellular and molecular mechanisms through which inositols improve insulin resistance are still poorly understood and remain largely to be determined.

An important issue is that inositol supplements exert a number of systemic effects that may significantly influence ovary function, regardless of the inositol amounts taken up by ovary. Following acute administration, both MI and DCI are distributed mainly to liver and kidney, as highlighted in studies with radiolabeled phosphatidylinositol (Shimizu et al., 2010). In liver, MI and DCI are incorporated into phospholipids and then conveyed by lipoproteins (HDL in particular) to all the tissue; notably, following single administration, free concentrations of MI and DCI rapidly decrease to very low levels (Shimizu et al., 2010). In terms of therapeutic effect, it seems that MI/DCI combinations might be more effective than either inositol species alone; however, the precise MI/DCI ratio to be used is a matter of debate. Available evidence is inadequate to provide a definite answer regarding the optimal MI:DCI ratio to be used as combination therapy, because specific pharmacokinetics data comparing MI, DCI and combinations are currently lacking. Moreover, given the importance of dosage on the effectiveness of each inositol species, the ratio between MI and DCI may be less important than the absolute concentrations of either MI or DCI (a given combination should include at least 300–1500 mg DCI and 2–4 g MI). Of note, most available pharmaceutical preparations based on MI/DCI combination provide very low amounts of DCI (13.8–27.6 mg), insufficient to achieve adequate levels, as predicted by studies showing DCI effects on glucose metabolism and insulin resistance.

In conclusion, based on the results from intervention studies, both treatments with MI and with DCI could be proposed as a potential valid therapeutic approach for the treatment of IR and its associated metabolic and reproductive disorders, such as those occurring in women affected by PCOS. Unfortunately, most studies reporting effects of inositol compounds on PCOS do not include other treatment groups so that direct comparisons with other available drugs are lacking. The chance is provided by two studies examining women treated with MI (4 g + 400 μg folic acid, daily) or metformin (1500 mg/day) (Raffone et al., 2010) and DCI (500 mg) or metformin (850 mg, both twice a day) (Piomboni et al., 2014). Despite the positive effects observed with all these treatments, the issue is, once again, the small populations examined that do not allow a detailed analysis of differences between groups. Further direct comparison studies are then necessary to face this limit. It has to be underlined, however, that efficacy of inositol compounds does not necessarily coincide with overcoming of reproductive failure, as reproductive endpoints were not included in a large part of the studies here examined. On the other hand, reproductive endpoints, whenever considered, were strongly affected by controlled ovary hyperstimulation rather than by inositols. In the absence of data showing the direct uptake by the ovary or the follicle of free MI or DCI present in the circulation following exogenous administration, any recommendation on MI:DCI optimal ratio to be administered seems arbitrary. Further preclinical and clinical studies are warranted to precisely define optimal doses and/or combination ratios.

## Author Contributions

All the authors have searched and analyzed the relevant literature, discussed with the co-authors the critical points and taken part in writing the manuscript. Furthermore, all the authors have read and approved the final (submitted) version of the present manuscript.

## Conflict of Interest Statement

The authors declare that the research was conducted in the absence of any commercial or financial relationships that could be construed as a potential conflict of interest.
